# Vaccinia Virus Induces Rapid Necrosis in Keratinocytes by a STAT3-Dependent Mechanism

**DOI:** 10.1371/journal.pone.0113690

**Published:** 2014-11-24

**Authors:** Yong He, Robert Fisher, Soma Chowdhury, Ishrat Sultana, Claudia P. Pereira, Mike Bray, Jennifer L. Reed

**Affiliations:** 1 Center for Biologics Evaluation and Research, Food and Drug Administration, Silver Spring, Maryland, United States of America; 2 Division of Clinical Research, National Institute of Allergy and Infectious Diseases, National Institutes of Health, Bethesda, Maryland, United States of America; University of Washington, United States of America

## Abstract

***Rationale*:**

Humans with a dominant negative mutation in STAT3 are susceptible to severe skin infections, suggesting an essential role for STAT3 signaling in defense against cutaneous pathogens.

***Methods*:**

To focus on innate antiviral defenses in keratinocytes, we used a standard model of cutaneous infection of severe combined immunodeficient mice with the current smallpox vaccine, ACAM-2000. In parallel, early events post-infection with the smallpox vaccine ACAM-2000 were investigated in cultured keratinocytes of human and mouse origin.

***Results*:**

Mice treated topically with a STAT3 inhibitor (Stattic) developed larger vaccinia lesions with higher virus titers and died more rapidly than untreated controls. Cultured human and murine keratinocytes infected with ACAM-2000 underwent rapid necrosis, but when treated with Stattic or with inhibitors of RIP1 kinase or caspase-1, they survived longer, produced higher titers of virus, and showed reduced activation of type I interferon responses and inflammatory cytokines release. Treatment with inhibitors of RIP1 kinase and STAT3, but not caspase-1, also reduced the inflammatory response of keratinocytes to TLR ligands. Vaccinia growth properties in Vero cells, which are known to be defective in some antiviral responses, were unaffected by inhibition of RIP1K, caspase-1, or STAT3.

***Conclusions*:**

Our findings indicate that keratinocytes suppress the replication and spread of vaccinia virus by undergoing rapid programmed cell death, in a process requiring STAT3. These data offer a new framework for understanding susceptibility to skin infection in patients with STAT3 mutations. Interventions which promote prompt necroptosis/pyroptosis of infected keratinocytes may reduce risks associated with vaccination with live vaccinia virus.

## Introduction

Vaccination against smallpox has long provided investigators with a simple way to study host responses to infection, by directly examining the spread of vaccinia virus in the skin. Although research on vaccination complications has traditionally focused on defects in humoral or cell-mediated immunity, there is increasing evidence that innate or acquired abnormalities of keratinocyte function may also result in uncontrolled virus spread. The failure of keratinocytes to provide an effective antiviral barrier appears to underlie the extensive infections which may occur when persons with skin disorders ranging from atopic dermatitis to burns and acne are vaccinated against smallpox [1].

One innate defect which was not known in the era of universal smallpox vaccination is the dominant negative mutation in the *STAT3* gene responsible for hyper-IgE (“Job's”) syndrome, which is characterized by a chronic eczema-like skin condition and enhanced susceptibility to cutaneous bacterial and viral infections, observed from days after birth and continuing throughout life [2]. There are no specific accounts of smallpox vaccine complications in hyper-IgE syndrome patients, but it seems likely that, as in naturally occurring herpesvirus and varicella infections, a defect in STAT3 signaling would permit extensive spread of vaccinia virus [3]–[4]. Defining a protective role of STAT3 in the response to infection might therefore lead to the development of novel countermeasures against vaccinia and other pathogens.

In the present study, we examine the role of STAT3 signaling in the response to smallpox vaccination, and show for the first time that it plays an essential role in the rapid programmed necrosis of keratinocytes induced by vaccinia virus. To focus on innate antiviral defenses, we inoculated severe combined immunodeficient (SCID) mice with ACAM-2000, the current licensed smallpox vaccine, and applied Stattic, a small-molecule inhibitor of both non-phosphorylated and phosphorylated STAT3 SH2 domains [5], to the vaccination site. In parallel studies, we measured viral replication, cell viability and inflammatory responses in ACAM-2000-infected human and mouse keratinocytes. We observed the effects of STAT3 inhibition via siRNA or Stattic, and the impact of blocking RIP1 kinase, an essential element in necroptosis, or caspase-1, which is required for pyroptosis [6]–[7]. Our data suggest that vaccinia rapidly triggers both necrosome and inflammasome activation in keratinocytes, resulting in marked suppression of viral replication and cell death, but these responses fail to occur in the absence of STAT3. Vero cells, which are known to be defective in some antiviral responses [8], permitted greater viral replication that was unaffected by the three inhibitors.

## Materials and Methods

### Cells, chemicals and reagents

HEK001 (ATCC, Manassas, VA) were maintained in Defined Keratinocyte Serum Free Medium (Life Technologies, Grand Island, NY) supplemented with 5 ng/ml recombinant EGF (Sigma, Saint Louis, MO). Murine 308 cells (provided by S. Yuspa, NCI, Bethesda, MD) and Vero cells (ATCC, Manassas, VA) were maintained in DMEM plus 10% fetal calf serum (Sigma, Saint Louis, MO). Primary epidermal keratinocytes grown at the air-liquid interface (Mattek, Boston, MA) were cultured according to manufacturer's instructions. A reporter plasmid encoding IFNβ promoter-luciferase (pNiFty3-Lucia) was purchased from Invivogen (San Diego, CA). Reporter plasmids encoding NFκB- and ISRE-luciferase, and control plasmid pRL-TK (*Renilla* luciferase) were obtained from Promega (Madison, WI). Lipofectamine 2000 was purchased from Life Technologies (Grand Island, NY). LPS, PGN and *S. typhimurium* flagellin were purchased from Invivogen (San Diego, CA). Antibodies to STAT3, TAK1, RIP1K, and β-Actin were purchased from Cell Signaling Technology (Danvers, MA). Species-specific HRP-conjugated secondary antibodies were purchased from Jackson Immunoresearch (West Grove, PA). STAT3 inhibitor Stattic and RIP1K inhibitor necrostatin-1 (Nec-1) were purchased from Santa Cruz Biotechnology (Santa Cruz, CA). Caspase-1 inhibitor Ac-YVAD-CMK and caspase-3 inhibitor Ac-DEVD-CHO were purchased from Cayman Chemical (Farmingdale, NY). Monoplex ELISA reagents for cytokine detection were obtained through R and D Systems (Minneapolis, MN). Bovine serum albumin (BSA) was purchased from Sigma (St. Louis, MO). Alamar Blue viable cell dye (Life Technologies, Grand Island, NY) and Cell Titer-Glo cell viability assay kit (Promega, Madison, WI) were used according to manufacturer's instructions. Scrambled control and STAT3-directed short interfering RNAs (ON-TARGET; Thermo Scientific, Pittsburgh, PA) were used according to manufacturer's instructions. Detailed information is provided in Methods S1.

### Mice

All experiments were approved by the Intramural Animal Care and Use Committee of the Center for Biologics Evaluation and Research, Food and Drug Administration and carried out in strict adherence to protocol (ASPReed11–12), including efforts to minimize suffering of study animals. Mice were housed and maintained according to NIH Animal Research Advisory Committee guidelines. Six to 12 week old SCID/NCr mice were obtained from the NCI Frederick Animal Production Program.

### Vaccinia virus strains and stock preparation

VACV-ACAM-2000 (Acambis, Inc., Cambridge, MA), a vaccine strain clonally derived from Dryvax, was obtained through the Centers for Disease Control. For virus stock preparations, Vero E6 cells (ATCC, Manassas, VA) were infected at a multiplicity of infection of 0.6 for 1 h at 37°C, then incubated at 37°C and monitored for a cytopathic effect daily. On day 3 postinfection, cells and cell culture supernatant were moved to centrifuge tubes and spun for 15 min at 2,000×g at 4°C in an Eppendorf 5404R centrifuge equipped with a swinging bucket rotor. The resulting pellets were processed with a chilled Dounce homogenizer, resuspended in cell culture medium, and clarified by centrifugation at 750×g for 10 min at 4°C. The supernatant was disrupted using a cup horn sonicator for 3 cycles each composed of 15 s at 90% power, 50% duty cycle, followed by 15 s on ice. Aliquots were removed to check sterility and viral titer, and the remainder was aliquoted and frozen at −70°C.

### Recombinant virus construction and purification

A recombinant vaccinia virus expressing luciferase (ACAM-luc) in addition to GFP to aid in clonal selection was constructed based on the ACAM-2000 (AY313847) strain of vaccinia. The protein trancripitonal unit for both luciferase and GFP expression was inserted at a truncated-host-range gene locus equivalent to the cowpox gene, CP77. A synthetic E/L vaccinia promoter was inserted into 5′ site of both the firefly luciferase gene and the GFP gene to drive expression during viral infection [9]. For CP77 specific recombination, the expression cassette was incorporated between two DNA flanked regions which correspond to sequence from 14000 to 14500 and 14581 to 15192 of ACAM-2000 genome. The full-length DNA constructs were chemically synthesized (Bio Basic Inc, Markham, ON, Canada), and incorporated into pUC57 plasmid to generate pUC ACAM-2000-luc-ΔCP77. Generation of the recombinant virus was performed as previously described [10]. In brief, Vero cells were infected with ACAM-2000 at an MOI of 0.2 pfu per cell and subsequently transfected with 3 µg of pUC ACAM-2000-luc-ΔCP77 using Lipofectamine 2000 (Life Technologies, Grand Island, NY) for 3 h. The cells were washed then cultured in DMEM supplemented with 10% FBS for 4 days. DNA from plaque-purified recombinant virus was purified from infected Vero cells using a QIAamp DNA mini kit (Qiagen, Valencia, CA). Nested PCR was carried out using the following primers: 5′ primer TTTCTCCTCGTTTGTCAATCATGTTAATG, corresponding to bases 14402–14430 of the ACAM0-2000 genome upstream of the CP77 locus, and 3′ primer GGTAGTAGGGTACTCGTGATTAATTTTATT, corresponding to bases 16004 to 16033 of the ACAM-2000 genome downstream of the CP77 locus. Gel electrophoresis analysis of the nested PCR reaction confirmed the presence of only one product, at the expected size of 4018 base pairs. The sequence of the insert was confirmed by DNA sequencing. Plaque-forming properties of ACAM-luc were similar to the parent ACAM-2000 strain (Methods S1).

### In Vivo STAT3 Inhibition and Scarification

A stock solution of the small molecule STAT3 inhibitor stattic was prepared in DMSO at 100 mM concentration. For topical dosing, mice were anesthetized with ketamine-xylazine and the fur was removed from the lower back using clippers. A 1% solution of stattic or an equal volume of vehicle alone was emulsified in Dermovan (Owen Laboratories, San Antonio, TX). This mixture was applied as a thin coating twice per day on the base of the tail. Scarification using 10^6^ pfu ACAM-2000 was performed as previously described [11].

### Plaque Assay

Skin biopsy tissues collected from euthanized mice were weighed and homogenized in cDMEM. The homogenized samples were sonicated for 3 cycles, each consisting of 15 s at 80% power at a 50% duty cycle and 15 s on ice, and were then serially diluted with cDMEM and plated onto confluent VeroE6 cells. After 1 h adsorption at 37°C, an overlay consisting of 1.5% methylcellulose in cMEM was added. Plates were incubated for an additional 3 days or until plaques were observed and were then stained with crystal violet in ethanol-formalin, and plaques were counted.

### Luciferase-Based Assay for Virus Growth

Vero cells or human or mouse keratinocyte cell lines (1×10^5^/well) in 12-well plates were incubated for 1 h at 37°C with a suspension of ACAM-luc at an MOI of 0.2 pfu per cell. Cells were washed to remove the viral inoculum, and fresh medium was added containing inhibitors or vehicle control. In initial experiments with Stattic, Nec-1, and caspase inhibitors, titration curves were performed to determine highest dose which yielded no change in cell viability over 48 hours (data not shown). Subsequent experiments were performed at 10 µM (Stattic), 20 µM (Nec-1), or 40 µM (caspase inhibitors). Primary human keratinocytes grown at the air-liquid interface were infected by applying ACAM-luc at an MOI of 20 pfu per cell to the top side of the culture well for 1 h at 37°C. Cells were washed to remove viral inoculum, and fresh medium containing inhibitors or vehicle control were added to the top side of the culture well. Cultures were continued for 24 hours for monolayer cultures or 48 hours for air-liquid interface cultures. Cells were harvested, lysed, and stored at −80°C for further testing. VeroE6 cells were seeded into 96-well plates (3×10^4^/well) and rested overnight. Cells were infected 1 h at 37°C with serial dilutions of viral samples. After overnight incubation, cells were harvested and lysed in 1×PLB solution (Promega, Madison, WI) at room temperature for 15 min. Luciferase activity was measured by a luciferase reporter assay kit (Promega, Madison, WI). A strong correlation was observed between luciferase activity and virus growth, as assessed by titration in Vero cells (Methods S1).

### Alamar Blue and Luciferase-Based Cell Viability Assays

Vero cells or human or mouse keratinocyte cell lines (1×10^5^/well) in 12-well plates were infected for 1 h at 37°C with ACAM-2000 at an MOI of 20 pfu per cell. Cells were washed to remove the viral inoculum, and fresh medium was added containing inhibitors or vehicle control. Cultures were continued for 6 to 24 hours. Conversion of Alamar Blue dye (Life Technologies, Grand Island, NY) to a reduced product was measured using a fluorescent platereader. In some experiments, loss of cell viability was quantified via evaluation of ATP, by adding luciferase and luciferin substrate to freshly lysed cells (Cell Titer-glo Kit, Promega, Madison, WI) according to manufacturer's instructions (Methods S1).

### ELISA

The detection and quantification of proinflammatory cytokines was performed using trioxsalen/UV-inactivated vaccinia infected cell culture supernatant, as previously described [12] with slight modifications. Briefly, trioxsalen (Sigma-Aldrich, St. Louis, MO) was added to a final concentration of 1 µg/ml, and the suspension was left at room temperature for 10 min. The trioxsalen-treated virus suspension was exposed to ultraviolet light for 15 min using a Stratalinker-1800 (Agilent Technologies, Santa Clara, CA). Virus inactivation was confirmed by tittering in Vero cells. TNF-α, IL-1β and IL-6 (R&D Systems; Minneapolis, MN) were measured using capture-detect ELISA systems according to manufacturer's recommendations.

### Reporter assays

Vero cells or human or mouse keratinocytes (2×10^4^/well) were transfected with 60 ng/well of firefly luciferase reporter plasmids, 10 ng/well of pTK-Renilla luciferase (pRL-TK, Promega) or 20 ng/well of pGL3-renilla luciferase prior to addition of virus or stimuli. Cell lysates were prepared for measurement of luciferase activity according to kit instructions.

### Transient RNA interference and transfections

STAT3 transcript was targeted in keratinocytes using a pool of 4 small short interfering RNAs (ON-TARGET Plus Smart Pool, Thermo, CO, USA). In parallel, a pool of 4 nontargeting siRNA was used as negative control. In control experiments, HEK001 cells were transfected with STAT3 or nontargeting siRNA using Lipofectamine 2000 for 48 hours. Knockdown of STAT3 mRNA by 90.6% was confirmed by quantitative RT-PCR (Methods S1). To evaluate effect of STAT3 siRNA targeting on ACAM-2000 growth, HEK001 were transfected with STAT3-directed or nontargeting siRNA for 48 hours, then infected with ACAM-2000 as described above.

### Immunohistochemistry

Formalin-fixed, paraffin-embedded skin tissues were sectioned and processed through xylene to remove paraffin. Heat-induced epitope retrieval was performed prior to immunohistochemistry (IHC). Primary antibody reactive with mouse STAT3 (Cell Signaling Technology, Danvers, MA) was used according to manufacturer recommendations. Primary antibodies were detected with biotinylated secondary antibodies (Jackson Immunoresearch, West Grove, PA) and streptavidin/horseradish peroxidase conjugate (GE Healthcare, Pitscataway, NJ) with peroxidase substrate (Sigma, St. Louis, MO).

### Immunoblot

HEK001 keratinocytes were incubated for 1 h at 37°C with a suspension of ACAM-2000 at 20 MOI. Cells were washed to remove the viral inoculum, and the culture was continued for 2 h at 37°C. Cells were collected in 4 volumes of ice-cold hypotonic buffer: 20 mM HEPES-KOH (pH 7.5), 10 mM KCl, 1.5 mM MgCl2, 1 mM Na EDTA, 1 mM Na EGTA, 0.1 mM AEBSF (all from Sigma, St. Louis, MO) and protease inhibitor cocktail (Roche, Indianapolis, IN). After incubation on ice for 30 min, cells were disrupted by 3 freeze-thaw cycles, followed by passage through a 22 gauge needle (Fisher, Pittsburgh, PA) 15 times. Cell lysates were centrifuged at 12,000 rpm for 30 minutes. Proteins in the collected supernatants were separated by electrophoresis using NuPAGE 4–12% protein gels and transferred onto nitrocellulose membranes (InVitrogen, Grand Island, NY). Dilutions of primary antibody (1∶200) and HRP-labeled secondary antibody (1∶1000) were performed in 5% milk blocking buffer. Visualization of reactive protein bands was performed using an enhanced chemiluminescence detection kit (GE Healthcare, Pitscataway, NJ). Quantification was performed using Image J software.

### Statistical analysis

Statistical analysis was performed using Prism 5 software (GraphPad Software, La Jolla, CA). Differences between groups were assessed by t-test, with statistical significance defined as p≤0.05.

## Results

### STAT3 is expressed by keratinocytes in vaccination lesions

**Figure 1:**
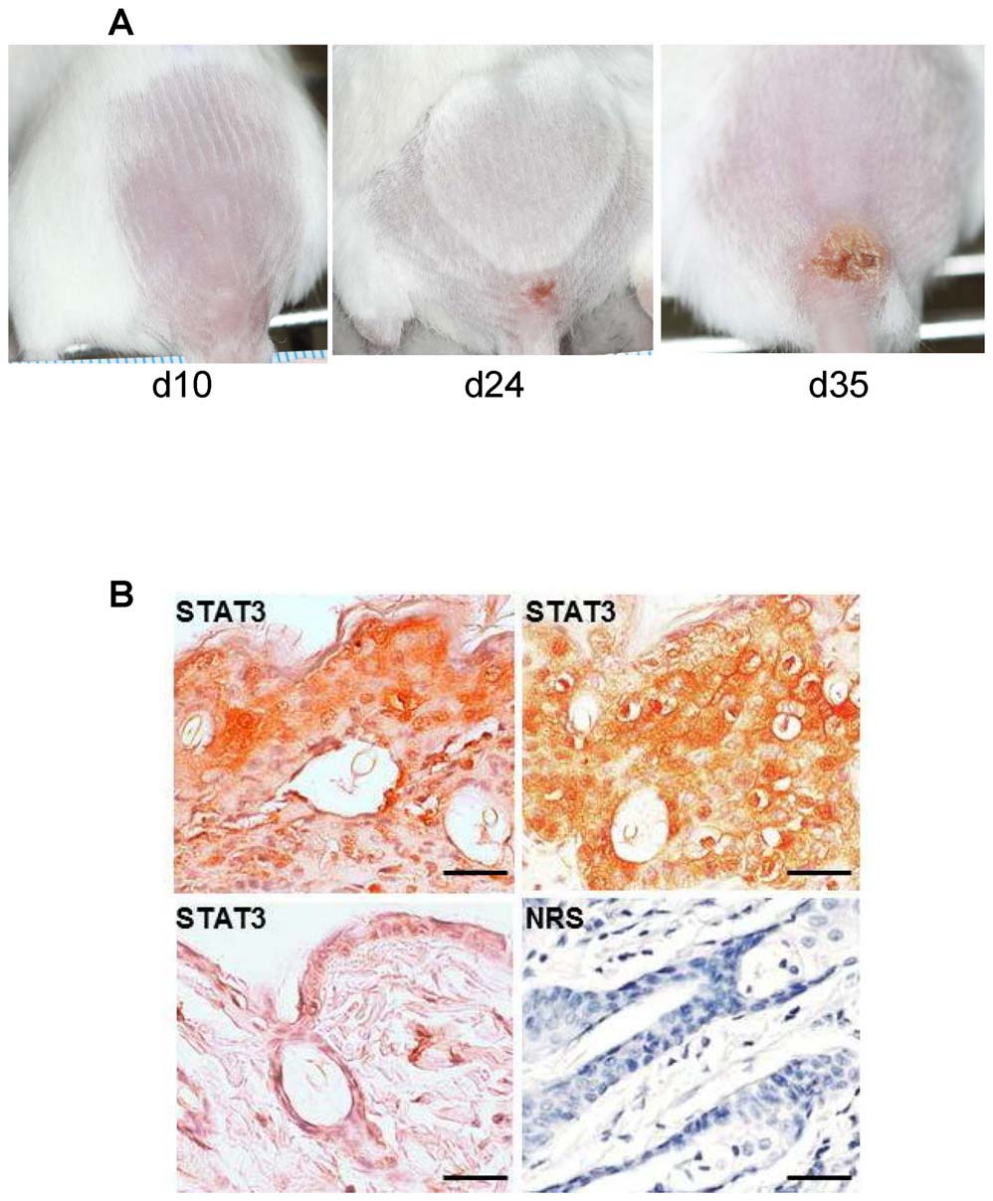
STAT3 detection in the primary vaccinia lesion of infected SCID mice. A) Slowly growing primary lesion in vaccinia infected SCID mice. B) STAT3 detected by immunohistochemistry in formalin fixed and paraffin embedded skin tissue from terminal vaccinia lesions of infected SCID mice (top panels) or uninfected SCID mouse (lower left). Lower right panel, normal rabbit serum negative control antibody detection in representative terminal vaccinia lesion. Scale bars, 100 µm.

SCID mice scarified at the base of the tail with ACAM-2000 develop an ulcer which gradually expands over 5–6 weeks (Figure 1A). Lesion formation is accompanied by progressive lethargy and cachexia, which are ultimately lethal. Histological analysis of late-stage lesions demonstrated marked expression of STAT3 in keratinocytes. STAT3 was partially localized to nuclei, as previously observed in healing wounds [13].

### Topical treatment with a STAT3 inhibitor causes more severe infection

**Figure 2:**
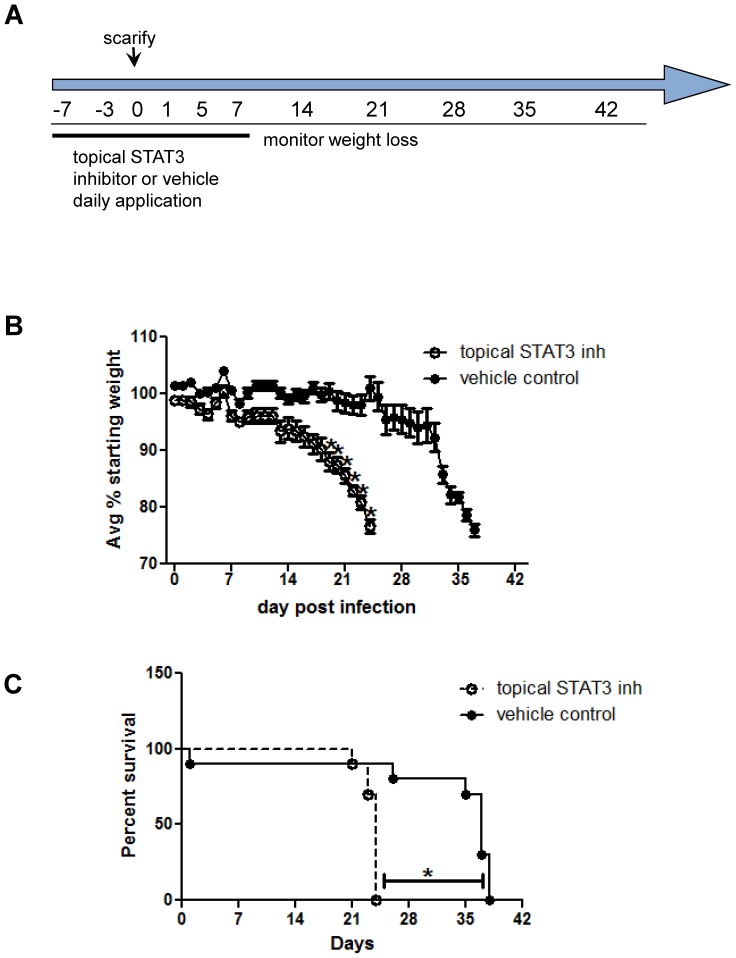
Topical application of Stattic accelerates vaccinia disease. A) Schedule of SCID mouse experiments, in which Stattic or DMSO was applied topically for one week before and after ACAM-2000 scarification. B) Weight loss in ACAM-2000 infected SCID mice treated with STAT3 inhibitor stattic or vehicle control. Mean of 9–10 mice is shown. C) Survival proportions in ACAM-2000 infected SCID mice treated with STAT3 inhibitor stattic or vehicle control (n = 9–10 per group). Asterisk, p≤0.05. Representative of three experiments is shown.

**Figure 3:**
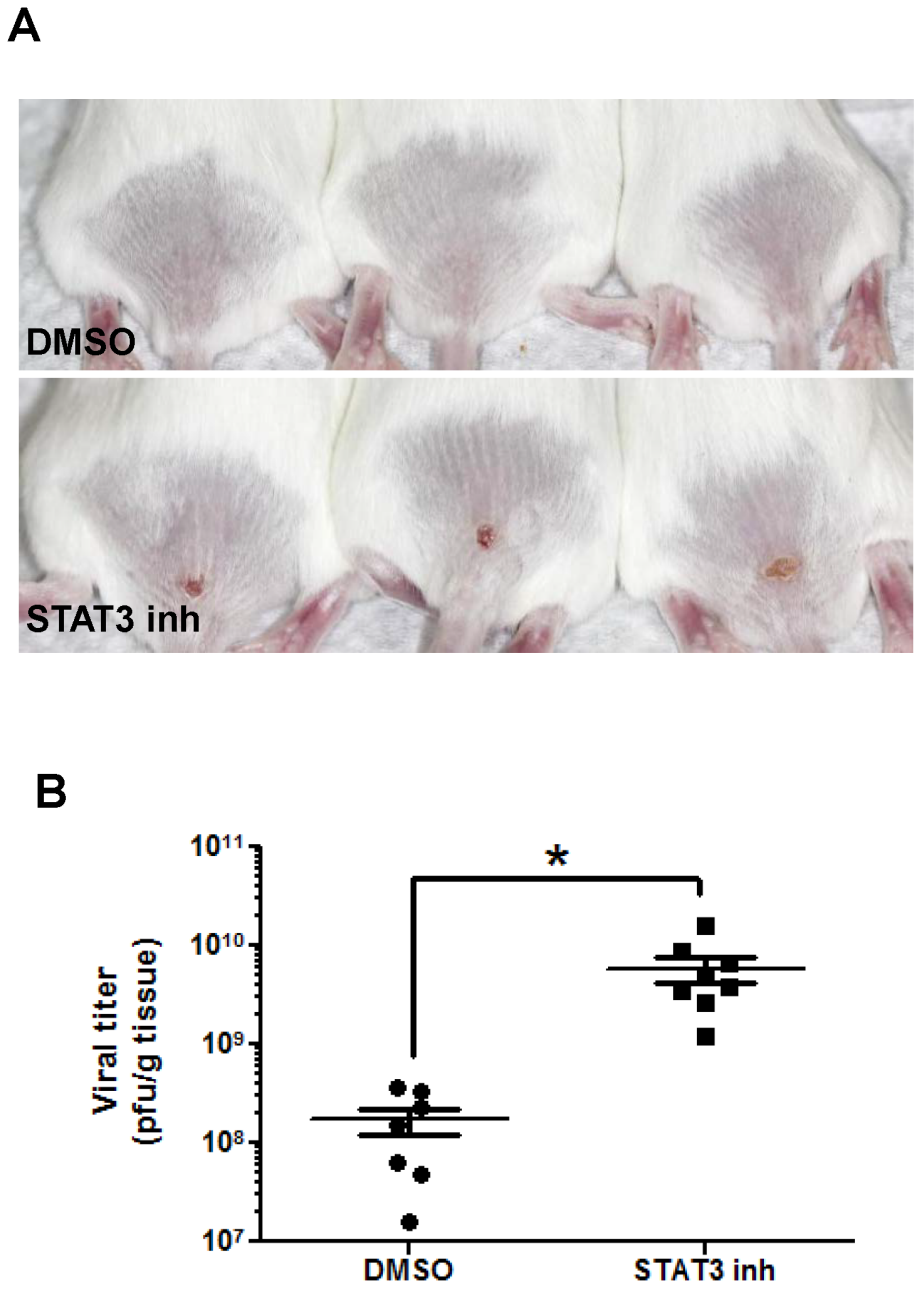
Topical STAT3 inhibition is associated with increased viral recovery. STAT3 specific inhibitor stattic or DMSO was applied topically, for one week before and continuing through one week after scarification. A) Representative lesions in DMSO (upper row) and stattic (lower row) treated mice were photographed on day 13, and virus recovery on day 13 (C) was assessed in homogenized primary lesion tissue (n = 7–8). Asterisk, p≤0.01.

The daily application of Stattic to the base of the tail for 7 days before and after scarification with ACAM-2000 (Figure 2A) resulted in a much earlier onset of weight loss and a shorter time to death than in mice treated with the DMSO vehicle (Figures 2B–2C). A subset of mice was euthanized at day 13, when vaccination lesions in control animals were barely visible, but were much larger in Stattic-treated mice (Figure 3A). Tissues from the lesion site showed a significantly higher titer of virus in treated mice (Figure 3B), indicating that inhibition of STAT3 impairs mechanisms by which keratinocytes control vaccinia virus infection.

### Vaccinia virus replicates to much lower titers in keratinocytes than in Vero cells

**Figure 4:**
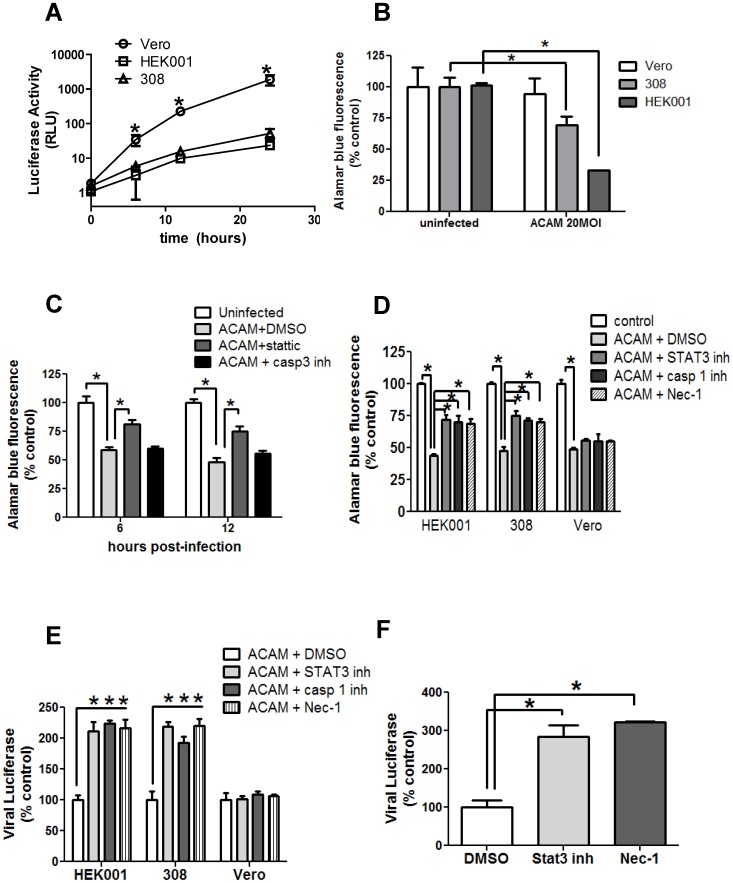
Inhibitors of STAT3, caspase-1 and RIP1K enhance vaccinia virus infection and preserve the viability of keratinocytes, but not Vero cells. A) Detection of luciferase activity over 24 hours post infection with ACAM-luc at 0.2 MOI in HEK001 and 308 keratinocytes, and Vero cells. Mean of replicate wells is plotted. B) Infection of keratinocytes (HEK001, 308) or Vero cells was performed with ACAM-2000 at 20 MOI. Viability at 6 hours post-infection was assessed by Alamar blue dye conversion (n = 4). C) Infection of HEK001 was performed with ACAM-2000 at 20 MOI, with vehicle control, Stattic, or caspase-3 inhibitor added. Viability at 6 and 12 hours post-infection was assessed by Alamar blue dye conversion (n = 4). D) Infection of keratinocytes (HEK001, 308) or Vero cells was performed with ACAM-2000 at 20 MOI, in the presence of DMSO vehicle control, STAT3 inhibitor, caspase-1 inhibitor, or Nec-1. Viability at 24 hours was assessed by Alamar blue dye conversion (n = 4). E) Keratinocytes (HEK001, 308) or Vero cells were infected with ACAM-luc at 0.2 MOI, in the presence of DMSO vehicle control, STAT3 inhibitor, Nec-1, or caspase-1 inhibitor. Luciferase activity at 24 hours is expressed as percent of uninfected control (n = 4). F) Differentiated primary keratinocytes at the air-liquid interface were infected with ACAM-luc at 20 MOI, in the presence of DMSO, STAT3 inhibitor, or Nec-1 (n = 2). Representative of triplicate experiments is shown. Asterisk, p≤0.05.

We made use of the murine epidermal cell line 308 and the human epidermal cell line HEK001 to further evaluate the role of STAT3 signaling in the response of keratinocytes to vaccinia virus. We generated ACAM-luc, a recombinant ACAM-2000 with a luciferase reporter gene under an early-late viral promoter, and used it to assess the level of viral replication in cultured cells. At a low multiplicity of infection (MOI) of 0.2, we found that ACAM-luc replicated more rapidly and to higher titers in Vero cells, a standard laboratory cell line, than in murine 308 cells, or the human HEK001 keratinocytes (Figure 4A).

### Vaccinia-infected keratinocytes die more rapidly than Vero cells

To more readily compare the effects of vaccinia virus infection on keratinocytes and Vero cells, we infected cells with ACAM-2000 at an MOI of 20 and measured cell viability by Alamar blue fluorescence. At 6 h post-infection, there was a significant loss of viability of 308 and HEK001 cells, while Vero cells showed little change (Figure 4B). A nearly identical loss of viability at 6 hours was observed measuring ATP release as an alternative readout (Methods S1; data not shown). The speed of the cell death response seemed inconsistent with apoptosis. In fact, inhibition of apoptosis using a specific caspase-3 blocker did not impact keratinocyte viability at 6 or 12 hours post-infection with ACAM-2000 at an MOI of 20 (Figure 4C). However, inhibition of STAT3 with Stattic (10 µm) increased cell viability at 6 and 12 hours.

### Inhibition of caspase-1, RIP1K or STAT3 maintains viability of infected keratinocytes

Vero, 308, and HEK001 cell lines all showed a similar loss of viability 24 hours after infection with ACAM-2000 at an MOI of 20 (Figure 4D). However, when infected 308 and HEK001 cells were treated with Stattic, or with an inhibitor of caspase-1, or an inhibitor of RIP1K (Nec-1), there was a significant increase in viability, suggesting that some of the vaccinia-infected keratinocytes were undergoing rapid inflammasome- or necrosome-mediated cell death. Inhibitor treatment of infected Vero cells produced no effect (Figure 4D).

### Inhibition of caspase-1, RIP1K or STAT3 permits increased viral replication in keratinocytes

In a parallel experiment to the above, HEK001, 308 and Vero cells were infected with ACAM-luc at an MOI of 0.2 and treated with Stattic or with an inhibitor of caspase-1, with Nec-1 or with vehicle control. All three inhibitors produced a marked increase in viral replication in the keratinocytes, but not in Vero cells (Figure 4E), suggesting that the rapid death of keratinocytes through inflammasome and necrosome signaling pathways limits virus growth. A similar result was obtained by titration of infectious ACAM-2000 in lysates of HEK001 cells, infected at 0.2 MOI and sampled at the 6 hour time point (60160±15707 pfu/ml in Stattic- treated cultures, versus 27680±12238 pfu/ml in DMSO-treated cultures). When primary human keratinocytes grown at an air-liquid interface were treated with Stattic or with Nec-1 and infected with ACAM-luc at an MOI of 20, virus replication measured by luciferase activity was markedly increased, compared to a vehicle control (Figure 4F).

### Stattic and Nec-1 treatment reduce inflammatory responses in infected keratinocytes

**Figure 5:**
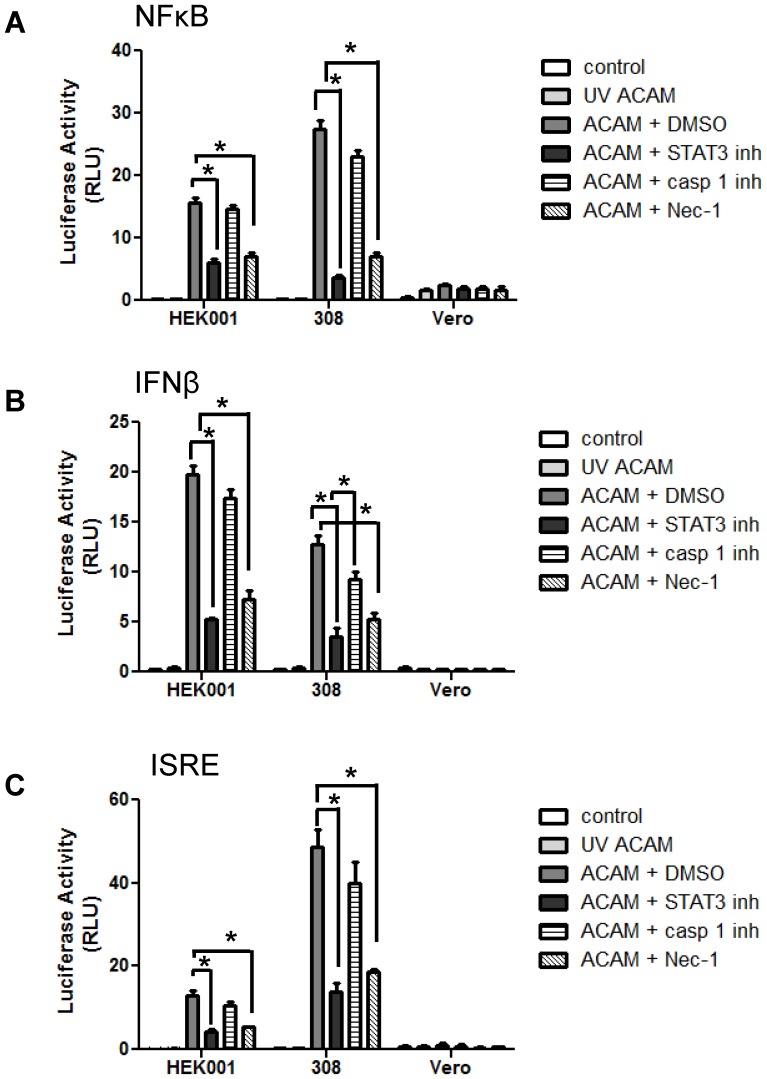
Inhibitors of STAT3 and RIP1K, but not caspase-1, suppress inflammatory responses to vaccinia virus infection in keratinocytes. HEK001 and 308 keratinocytes and Vero cells were transfected with reporter plasmids encoding luciferase under the control of promoters for NFκB (A), IFNβ (B), or ISRE (C). Cells were then challenged with UV inactivated ACAM-2000, or with infectious ACAM-2000 at 20 MOI combined with DMSO, Stattic, caspase-1 inhibitor, or Nec-1. Luciferase activity was detected at 24 hours post-infection (n = 4 wells per condition). Representative of triplicate experiments is shown. Asterisk, p≤0.05.

Our initial results suggested that infected keratinocytes can die through a STAT3-dependent inflammatory pathway. Because acute pro-inflammatory responses to vaccinia virus are regulated by NF-κB and type I interferon production, we studied the impact of STAT3 inhibition on these signaling pathways. Keratinocytes were transfected with reporter plasmids directing luciferase expression under the control of NF-κB, IFN-β, or ISRE promoter elements, then infected with wild-type ACAM-2000. Increased NF-κB (Figure 5A), IFN-β (Figure 5B), and ISRE (Figure 5C) promoter activity was observed in ACAM-2000 infected cells, consistent with acute antiviral responses. No activity was seen in keratinocytes that remained uninfected or were exposed to UV-inactivated ACAM-2000, and only trace to absent responses were seen in Vero cells. When ACAM-2000-infected keratinocytes were treated with Stattic or with Nec-1, there was a significant reduction in all three responses. In contrast, the caspase-1 inhibitor was less effective in reducing NFκB, IFN-β and ISRE promoter activity in ACAM-2000-infected keratinocytes.

### siRNA targeting STAT3 mimics Stattic effects

**Figure 6:**
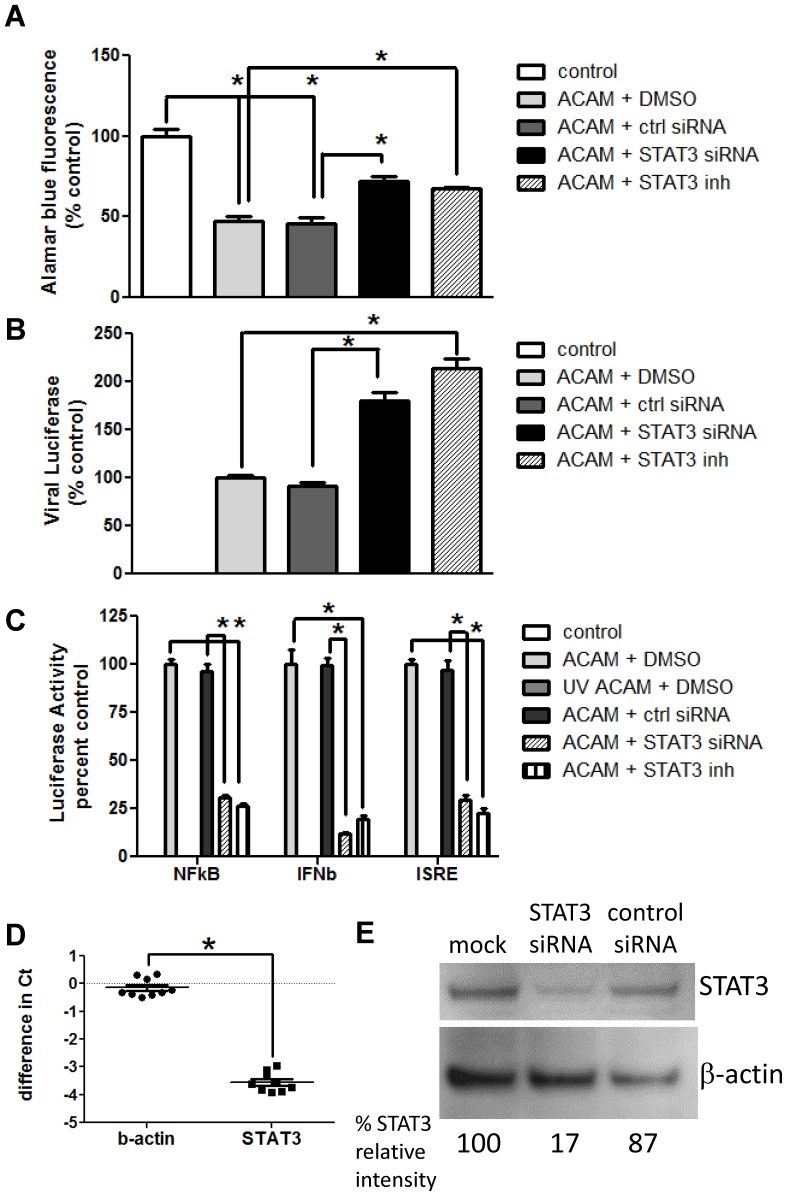
Treatment of infected keratinocytes with STAT3-specific siRNA partially preserves cell viability and increases virus replication and inflammatory responses. HEK001 cells were transfected with control or STAT3-specific siRNA preparations for 48 hours or treated with DMSO or Stattic immediately prior to infection. A) Wells were infected with ACAM-2000 at 20 MOI. Viability at 24 hours was assessed with Alamar blue fluorescence (n = 4). B) Wells were infected with ACAM-luc at 0.2 MOI. Cells were harvested at 24 hours, and luciferase in cell lysates was detected (n = 4). C) Cells were transfected with reporter plasmids encoding luciferase downstream of NFκB, IFNβ, or ISRE promoter elements. Transfected cells were infected with ACAM-2000 at 20 MOI, or with UV inactivated ACAM-2000. Luciferase signal was measured at 24 hours postinfection (n = 4). Representative of triplicate experiments is shown. D) HEK001 cells were transfected with scrambled control siRNA, or STAT3 directed siRNA. Cells were harvested at 48 hours and total mRNA and cDNA were prepared. Transcripts for -actin and STAT3 were quantified using qRT-PCR. Difference in threshold cycle number (Ct) between cells treated with control and STAT3 directed siRNA is shown (n = 9). E) HEK001 cells were mock transfected, or transfected with scrambled control siRNA or STAT3 directed siRNA. Cells were harvested at 48 hours. STAT3 and β-actin were detected in whole cell lysates by immunoblot. Relative intensity of STAT3 band, normalized to β-actin control band, was calculated using densitometric analysis for each treatment group. Representative of duplicate experiments is shown. Asterisk, p≤0.05.

To exclude the possibility that keratinocyte responses might reflect off-target effects of Stattic, we used a parallel approach, transfecting HEK001 cells with STAT3-directed siRNA prior to vaccinia infection. As seen with Stattic, STAT3-directed siRNA treatment resulted in both increased viral replication in cells infected with ACAM-luc, and increased cell viability in cells infected with ACAM-2000, compared to cells transfected with the scrambled siRNA control (Figure 6A–B). STAT3-targeted siRNA also reduced signals from NF-κB, IFN-β and ISRE promoter-driven reporter constructs in ACAM-2000-infected keratinocytes (Figure 6C). Knockdown of STAT3 with specific siRNA in HEK001 keratinocytes was calculated at greater than 90% at the transcript level, and greater than 80% at the protein level (Figure 6D–E).

### Stattic treatment reduces inflammatory responses to TLR ligands

**Figure 7:**
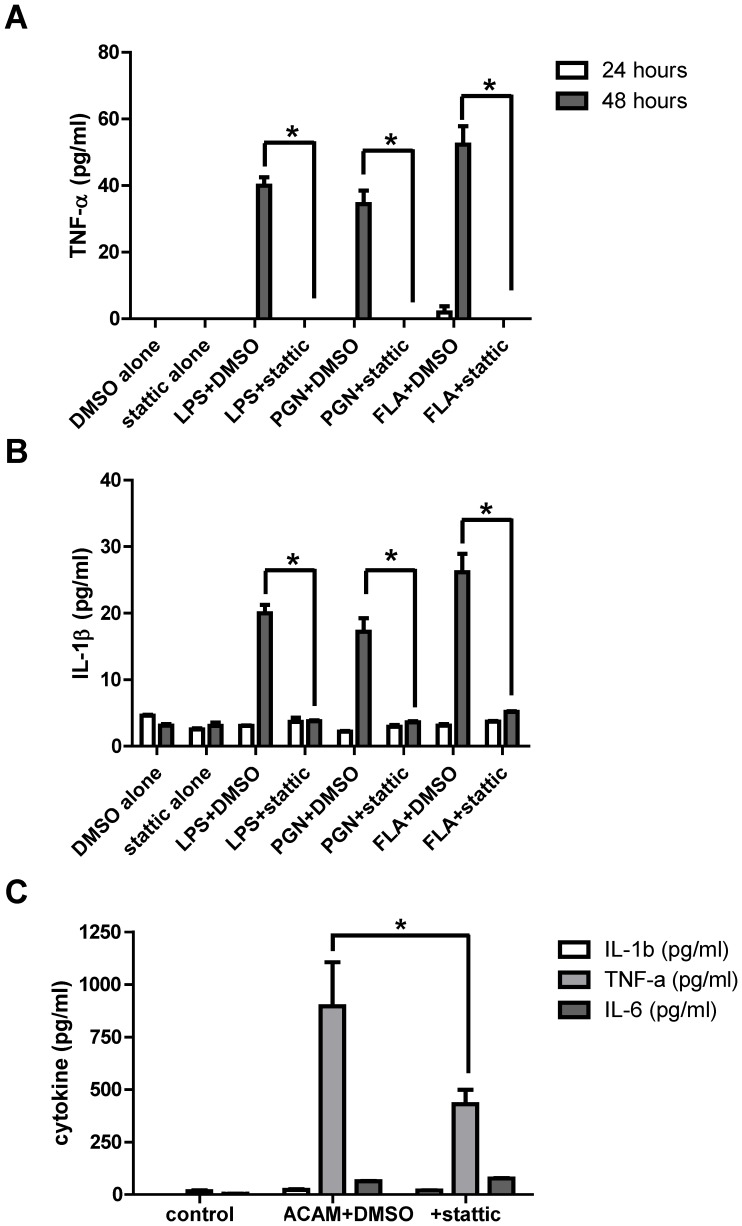
Treatment with Stattic reduces inflammatory responses in keratinocytes exposed to TLR ligands. HEK001 cells were pretreated with Stattic or vehicle, then challenged with bacterial lipopolysaccharide (LPS), peptidoglycan (PGN) or *S. typhimurium* flagellin (FLA). TNF-α (A) and IL-β (B) in supernatants was assessed by ELISA at 24 and 48 hours. C) HEK001 cells treated with vehicle or Stattic were infected with ACAM-2000 at MOI  = 20. Secreted TNF-α, IL-1β, and IL-6 were assessed at 12 hours (n = 2 wells per condition). Representative of triplicate experiments is shown. Asterisk, p≤0.05.

When HEK001 cells were pretreated with Stattic and exposed overnight to bacterial lipopolysaccharide (LPS), bacterial peptidoglycan or *S. typhimurium*-derived flagellin, they showed markedly diminished secretion of the inflammatory mediators TNF-α and IL-1β, (Figures 7A–B) and of IL-6 (data not shown), compared to vehicle-treated cells. In HEK001 cells infected with ACAM-2000, TNF-α was the major cytokine detected at the 24-hour time point. Pretreatment with Stattic significantly reduced TNF-α secretion, compared to vehicle control (Fig. 7C).

### Mechanism of Action of Stattic in Keratinocytes

**Figure 8:**
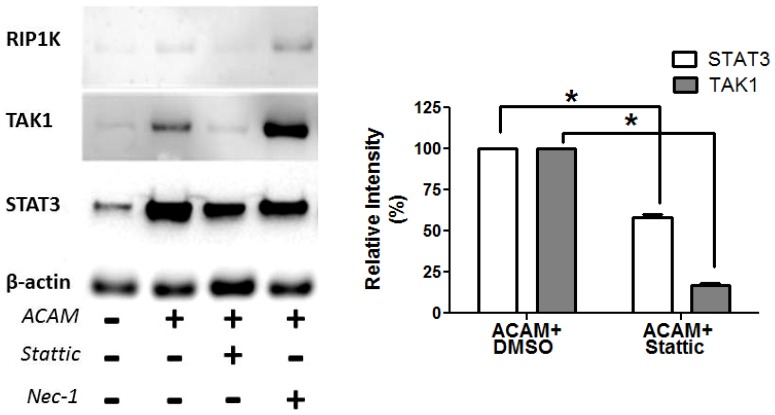
Stattic reduces STAT3 and TAK1 detection in cytosol of infected keratinocytes. Left: HEK001 keratinocytes were uninfected, or infected for 3 hours with ACAM-2000 at 20 MOI, in the presence of DMSO, Stattic, or Nec-1. At 3 hours cells were collected in cold hypotonic buffer. Left: Cytoplasmic fractions were evaluated for STAT3, TAK1, RIP1K, and housekeeping protein β-actin by immunoblot. Right: Relative intensity of STAT3 and TAK1 protein bands in infected, Stattic-treated wells, compared with infected, DMSO treated wells (n = 2 experiments). Asterisk, p≤0.05.

Early events in vaccinia infection of keratinocytes have not been extensively evaluated. To help understand possible contributions of STAT3 to early antiviral signaling, HEK001 keratinocytes were harvested at 3 hours post-infection with ACAM-2000 at 20 MOI, and hypotonic lysates were prepared for immunoblot analysis (Figure 8). Compared with β-actin control protein, detection of both STAT3 and the innate signaling kinase TAK1 were increased in cytoplasmic fractions of infected cells (Figure 8). The addition of STAT3 inhibitor Stattic, but not RIP1K inhibitor Nec-1, significantly reduced cytoplasmic detection of both STAT3 and TAK1 in infected cells (Figure 8).

## Discussion

The goal of these studies was to illuminate intrinsic antiviral resistance of keratinocytes, which within minutes to hours after vaccinia infection undergo pro-inflammatory suicide. Our results provide new insights into the complexity of multi-layered resistance against skin pathogens. Effective defense appears to require a combination of innate responses of keratinocytes, to restrict pathogen replication and alert the immune system, in addition to adaptive immune responses, to limit pathogen dissemination and kill infected cells. Our data suggest that rapid necrosis of infected cells eliminates an essential niche for virus replication, and establishes an antiviral firewall in part through paracrine NFκB, TNF-α, and IFN-β signals. Since the host response of programmed necrosis has evolved through natural selection, it possibly also enhances protective adaptive immune responses to virus infection, in ways that have not yet been fully elucidated. Together these observations highlight for the first time host-protective roles for keratinocyte programmed necrosis, which in previous studies had only been linked with pathogenic cutaneous inflammation [14].

Rapid programmed cell death in response to vaccinia virus was earlier demonstrated by Cho and colleagues, who inoculated mice intraperitoneally with the virulent WR strain [15]. In their study, RIP3 deficient mice infected with VACV-WR via intraperitoneal route had increased virus titers, greater mortality, and reduced inflammation compared with similarly infected wild type mice. The authors concluded that damage-associated molecular patterns released from sites of necroptosis help to control the spread of infection by eliciting a strong inflammatory response. Our data confirm and extend these observations, demonstrating for the first time that keratinocytes targeted by the vaccine strain ACAM-2000 undergo rapid cell death dependent on both caspase-1 (pyroptosis) and RIP1K (necroptosis). Blocking either of these early cell death responses increased vaccinia replication in vitro, highlighting contributions of both pathways in limiting viral replication. The current data additionally point to RIP1K-dependent signaling pathways, but not caspase-1, as critical activators of NFκB and type I interferon in keratinocytes. More work will be required to evaluate the relative roles of receptors such as TNFR, TLRs, and viral nucleic acid sensors (e.g. NALP1, RIG-I, DAI) in triggering RIPK dependent keratinocyte death, and how vaccinia-encoded factors may subvert the necroptotic process, as has been described in cells infected with murine cytomegalovirus [16].

Our studies are the first to our knowledge to indicate a host-protective role for STAT3 in virus-infected cells. In normal and in transformed cells, STAT3 promotes transcription of proteins which enhance cell cycle progression and antagonize apoptotic cell death [17]. In previous reports, signals from transcriptionally active STAT3 dimers were shown to keep virus-infected cells alive and promote pathogenesis [18]. Our work has incorporated several key differences from previous studies. First we focused on keratinocytes, rather than fibroblasts or other cell lines in which innate antiviral responses are either untested or known to be deficient. We also evaluated time points very early after in vitro and in vivo infection, and utilized STAT3 inhibitory approaches that block unphosphorylated and phosphorylated STAT3 [2], not just Tyr-705 activated monomers and dimers. Using this approach, we found rapid necrosis of vaccinia-infected keratinocytes, and cytoplasmic accumulation of STAT3 and TAK1, within a few hours post-infection. These finding prompt a consideration of new mechanisms by which STAT3 might aid detection and elimination of vaccinia in infected keratinocytes. STAT3 may facilitate association of innate signaling complexes involving TAK1, as was previously described for the IL6R gp130 complex [19]. A signaling scaffold function for STAT3 could result in prolonged association of the TAK1:RIP1K complex, which triggers necroptosis in studies of TNF-stimulated fibroblasts [20]. Evaluating possible co-association of STAT3 with TAK1 with molecular sensors of infection is a focus of our ongoing studies. Alternatively or in addition, interactions between cytosolic STAT3 and mitochondrial proteins such as Complex I component Grim19 [21]–[22] could be essential for early antiviral functions requiring superoxide generation. We are currently evaluating in the keratinocyte system whether cytoplasmic STAT3 might enhance superoxide production, which in other cell types optimizes inflammasome activation [23] and may be essential for necroptosis as well [24]–[25].

The role of STAT3 in keratinocyte defense is not restricted to viral infection, as Stattic treatment also prevented keratinocytes from responding to TLR ligands associated with bacterial infection. Similar results were reported by Samavati and colleagues, using Stattic and STAT3-directed siRNA approaches in LPS stimulated macrophages [26]. Dampened inflammatory responses to bacterial triggers in the absence of STAT3 signaling could be consistent with the clinical features of Job's syndrome, in which patients may carry large bacterial burdens in the absence of fever, malaise, and other symptoms normally induced by cytokines [2]. Our future studies will evaluate the possible role of STAT3 in amplifying early responses to cutaneous bacterial infection, and how these innate responses may be linked with appropriate T-helper lymphocyte activation and function. Despite eradication of naturally occurring smallpox, potential use of variola virus as a bioterrorism agent has spurred continued use of the licensed smallpox vaccine (ACAM-2000) among designated first responders and military personnel [27]. ACAM-2000 elicits poxvirus-directed T lymphocyte responses and neutralizing antibodies in animal models, suggesting vaccination could confer protection against variola [28]. However vaccination in susceptible people is associated with a risk of virus dissemination from the inoculation site. Eczema vaccinatum (EV), a rare but life-threatening complication of smallpox vaccination, features a rapidly progressing rash over a large proportion of the skin surface in a subset of patients with AD/eczema and other skin disorders [1]. Our current data suggest that immediate responses to vaccinia infection in the skin are probably important in determining susceptibility to severe outcomes like EV. With this in mind, improved future treatments for EV might include approaches which bolster cutaneous antiviral defense. Our future studies will test topical small molecule approaches to accelerate death of vaccinia infected keratinocytes, using a model of inducible keratinocyte specific STAT3 deficiency.

In summary, our data provide evidence for STAT3-dependent necroptosis and pyroptosis in keratinocytes, contributing to anti-vaccinia defense of the skin. The data may point to novel roles for STAT3 in facilitating rapid responses to cutaneous infection and damage. Understanding STAT3 dependent innate signaling mechanisms may offer new means of identifying individuals susceptible to severe skin infection, and new opportunities for their treatment.

## Supporting Information

Methods S1 **Additional details regarding validation of ACAM-luc in vitro infection parameters, siRNA studies, and measurement of cell viability in vitro are provided in Methods S1.**
(PDF)
