# Supplementary material for: Vaccinia Virus Induces Rapid Necrosis in Keratinocytes by a STAT3-Dependent Mechanism
Source: PLoS One. 2014 Nov 24;9(11):e113690. doi: 10.1371/journal.pone.0113690 (PMC4242661; doi:10.1371/journal.pone.0113690)
Supplement: Methods S1 — Additional details regarding validation of ACAM-luc in vitro infection parameters, siRNA studies, and measurement of cell viability in vitro are provided in Methods S1. (PDF) [file pone.0113690.s001.pdf]

Supplemental Methods

Validation of ACAM-luc in vitro infection properties: Plaque Forming Capability of ACAM-luc

To confirm plaque-forming properties of ACAM-luc were similar to the parental strain ACAM-2000, titration of both strains was performed side-by-side in BSC40, Vero, and murine keratinocyte 308 cells. Cells were plated in 48 well dishes at $1x10^5$ cells/well. Infection was performed with 2000 pfu/well followed by 7 sequential 3-fold dilutions across the plate. After 1 hour at 37^oC, the viral inoculum was removed, and cells were overlaid with methylcellulose as described in Materials and Methods. Cultures were continued for 72 hours, then stained with crystal violet for assessment of plaques.

Validation of ACAM-luc in vitro infection properties: Relationship between luciferase activity and plaque number

Confluent Vero cells in a 48-well culture dish were inoculated with ACAM-luc at 2000 pfu/ml, and 7 consecutive 3-fold dilutions were performed. Incubation proceeded for 1 hour at 37^oC, and the inoculum was removed. In some wells, fresh medium was added and the cultures were continued for 12 hours, followed by harvest of cells for evaluation of luciferase activity as described in Materials and Methods. Other wells were covered with methylcellulose overlay, and incubation was continued for 72 hours. Evaluation of plaques was performed via crystal violet staining as described in Materials and Methods. Plaque numbers and luciferase activity in replicate wells were plotted with correlation analysis.

Inhibition of STAT3 with siRNA

The sequences included in the STAT3-directed siRNA pool were:

5’CGUUAUAUAGGAACCGUAA3’, 5’UUACGGUUCCUAUAUAACG3’;

5’GGAGAAGCAUCGUGAGUGA3’, 5’UCACUCACGAUGCUUCUCC3’; 5’CCACUUUGGUGUUUCAUAA3’, 5’UUAUGAAACACCAAAGUGG3’; 5’UCAGGUUGCUGGUCAAAUU3’, 5’AAUUUGACCAGCAACCUGA3’.

Primers for detection of STAT3 by RT-PCR were: 5’GAGAAGGACATCAGCGGTAAG3’ (forward) and 5’CAGTGGAGACACCAGGATATTG3’ (reverse).

Primers for detection of β-actin by PCR were 5’GGACCTGACTGACTACCTCAT3’ (forward) and 5’CGTAGCACAGCTTCTCCTTAAT3’ (reverse).

To determine efficiency of knockdown, HEK001 cells were transfected with scrambled control siRNA, or STAT3 directed siRNA. After 48 hours cells were harvested to prepare total mRNA and cDNA. Transcripts for β-actin housekeeping gene or STAT3 were quantified using qRT-PCR. The threshold cycle number was expressed normalized to β-actin. Percent knockdown was calculated using the ΔΔCT method (n=9).

Cell Viability Measurement by Complementary Methods.

HEK001 human keratinocytes were untreated, or infected with unlabeled ACAM-2000 at 20 MOI in the presence of DMSO vehicle or 10 uM STAT3 inhibitor Stattic. Viability was measured using two complementary methods. In some wells, cells were freshly lysed (Cell Titer-Glo lysis reagent) immediately prior to addition of luciferase and luciferin substrate. Luminescence was measured immediately in 4 replicate wells. In other wells, Alamar blue dye reagent was added. Conversion to a fluorescent product was measured using a fluorescence plate reader in 4 replicate wells.
